# Serum C-reactive Protein Level Predicts Overall Survival for Clear Cell and Non-Clear Cell Renal Cell Carcinoma Treated with Ipilimumab plus Nivolumab

**DOI:** 10.3390/cancers14225659

**Published:** 2022-11-17

**Authors:** Yusuke Yano, Takaya Ohno, Kazumasa Komura, Wataru Fukuokaya, Taizo Uchimoto, Takahiro Adachi, Yosuke Hirasawa, Takeshi Hashimoto, Atsuhiko Yoshizawa, Shogo Yamazaki, Satoshi Tokushige, Kazuki Nishimura, Takuya Tsujino, Keita Nakamori, Shutaro Yamamoto, Kosuke Iwatani, Fumihiko Urabe, Keiichiro Mori, Takafumi Yanagisawa, Shunsuke Tsuduki, Kiyoshi Takahara, Teruo Inamoto, Jun Miki, Takahiro Kimura, Yoshio Ohno, Ryoichi Shiroki, Haruhito Azuma

**Affiliations:** 1 Department of Urology, Osaka Medical and Pharmaceutical University, 2-7 Daigaku-machi, Takatsuki City 569-8686, Japan; 2 Department of Urology, The Jikei University School of Medicine, 3-25-8 Nishi-shimbashi, Minato-ku, Tokyo 105-8461, Japan; 3 Department of Urology, Tokyo Medical University, 6-7-1 Nishi-shinjuku, Shinjuku-ku, Tokyo 160-0023, Japan; 4 Department of Urology, Fujita-Health University School of Medicine, 1-98 Dengakugakubo, Kutsukake, Toyoake 470-1192, Japan

**Keywords:** ipilimumab, nivolumab, renal cell carcinoma, C-reactive protein

## Abstract

**Simple Summary:**

Elevated serum C-reactive protein (CRP) level is one of the most established markers of systemic inflammation, potentially affecting tumor immune-microenvironmental status. Thus, we assessed the predictive value of serum CRP level for metastatic renal cell carcinoma (mRCC) treated with first-line ipilimumab and nivolumab using our real-world clinical dataset including non-clear cell RCC (nccRCC). Treatment record of 74 patients treated with ipilimumab and nivolumab for intermediate or poor-risk RCC defined by IMCD (international metastatic RCC database consortium). The one-year overall survival (OS) rate and objective response rate were 65% and 41% for all 74 mRCC patients, respectively. The receiver operating characteristic curve identified 1.0 mg/dL of serum CRP level as an ideal cut-off for predicting overall survival (OS). OS for patients with CRP > 1 mg/dL was significantly shorter than those with CRP < 1 mg/dL in both ccRCC (58 patient: *p* = 0.009) and nccRCC (16 patients: *p* = 0.008). The present study suggested that serum CRP level is a prognostic indicator for OS.

**Abstract:**

Serum C-reactive protein (CRP) is known to be a biomarker for systemic inflammatory reactions. In the present study, we sought to measure the predictive value of serum CRP level for metastatic renal cell carcinoma (mRCC) treated with first-line ipilimumab and nivolumab using our real-world clinical dataset including non-clear cell RCC (nccRCC). The clinical record of patients who underwent the first-line ipilimumab plus nivolumab treatment for mRCC including ccRCC and nccRCC from 2018 to 2021 was retrospectively analyzed. All patients were diagnosed with either intermediate or poor-risk group defined by IMCD (international metastatic RCC database consortium). In total, 74 patients were involved. The median age was 68 years and 24 (32.4%) patients deceased during the follow-up. Forty-five (61%) and 29 (39%) patients were classified into intermediate and poor-risk groups. The one-year overall survival (OS) rate and objective response rate were 65% and 41% for all 74 mRCC patients, respectively. The receiver operating characteristic curve identified 1.0 mg/dL of serum CRP level as an ideal cut-off for predicting overall survival (OS). Serum CRP > 1.0 mg/dL and nccRCC were the independent predictors for OS in 74 mRCC patients. OS for patients with CRP > 1 mg/dL was significantly shorter than those with CRP < 1 mg/dL in both ccRCC (58 patient: *p* = 0.009) and nccRCC (16 patients: *p* = 0.008). The present study indicated that serum CRP level is a prognostic indicator for OS in both ccRCC and nccRCC patients treated with the first-line ipilimumab plus nivolumab treatment.

## 1. Introduction

In the last decade, the treatment strategy for metastatic renal cell carcinoma (mRCC) has drastically changed with the emergence of immune checkpoint inhibitors (CPIs), such as agents targeting programmed cell death 1 (PD-1) and cytotoxic T-lymphocyte-associated protein 4 (CTLA-4). Several combination regimens with CPIs and tyrosine kinase inhibitors (TKIs) are now delivered as the first-line treatment for mRCC patients [1,2,3,4]. A regimen using PD-1 inhibitor (nivolumab) and CTLA-4 inhibitor (ipilimumab) is currently the only regimen of combined immunotherapy for the first-line treatment in mRCC patients with the International Metastatic Renal Cell Carcinoma Database Consortium (IMDC) intermediate and poor-risk groups, approved by the results from the randomized phase 3 Checkmate 214 trial [5]. The Checkmate 214 trial enrolled 1096 clear cell RCC (ccRCC) and exhibited approximately 75% of disease control rate (DCR), which was relatively modest compared to the other combination regimens using CPIs and TKIs. Thus, a reliable marker to predict the clinical effect of ipilimumab and nivolumab is urgently needed.

Recently, a biomarker analysis from the Checkmate 214 trial revealed that putative biomarkers previously reported to benefit from immune checkpoint inhibitor-containing regimens in mRCC, including PD-L1 expression on tumor cells and tumor mutation burden, were not predictive for survival in patients with mRCC treated with ipilimumab plus nivolumab, whereas their transcriptome analysis showed an association between inflammatory response and progression-free survival with nivolumab plus ipilimumab [6]. To date, a volume of efforts to delineate the risk prediction have been analyzed using putative indicators for systemic inflammatory response including neutrophil count, lymphocyte count, platelet count, and hemoglobin [7,8,9]. Particularly, serum C-reactive protein (CRP) is known to be a biomarker for systemic inflammatory reaction. A number of studies indicated the utility of serum CRP level as a prognostic and predictive biomarker for surgery, molecular targeted therapy, and the effect of CPIs in RCC [10,11,12,13]. In the present study, we sought to measure the predictive value of serum CRP level for mRCC treated with first-line ipilimumab and nivolumab using our real-world clinical dataset including non-clear cell RCC (nccRCC).

## 2. Materials and Methods

This retrospective study was designed using a multi-institutional dataset from Osaka Medical and Pharmaceutical University (Osaka, Japan), Tokyo Medical University (Tokyo, Japan), Fujita-Health University School of Medicine (Aichi, Japan), and the Jikei University School of Medicine (Tokyo, Japan). The project was approved by the Institutional Review Board (IRB) of the principal institution (Osaka Medical and Pharmaceutical University; approval number: RIN–750–2571). The study was performed in adherence to the principles of the World Medical Association Declaration of Helsinki. A form of written informed consent was acquired from each individual at the enrollment of the study. The clinical record was retrospectively queried. Inclusion criteria were patients who underwent first-line ipilimumab plus nivolumab treatment for metastatic renal cell carcinoma including ccRCC and nccRCC from 2018 to 2021. Patients previously treated with any anti-cancer agents or without the completion of blood examination prior to the first-line treatment were excluded from the study.

All patients were diagnosed with either intermediate or poor-risk group defined by IMCD (international metastatic RCC database consortium) risk groups [14]. Patient demographics included age, sex, body mass index (BMI), International Metastatic RCC Database Consortium (IMDC) risk score, prior nephrectomy before the first-line treatment, histology (clear cell carcinoma and non-clear cell carcinoma), location of the metastatic sites, the occurrence of immune-related adverse event (irAE), and the blood examination at the initiation of ipilimumab plus nivolumab (C-reactive protein: CRP, platelet-lymphocyte ratio: PLR, corrected calcium level, hemoglobin level, and neutrophil-lymphocyte ratio; NLR). The primary endpoints in the present study were overall survival (OS) and cancer-specific survival (CSS). OS and CSS were calculated as the interval from the initiation of the first-line treatment to the date of last follow–up or deaths from any cause (OS) and cancer-specific deaths (CSS). The secondary endpoint was objective response rate (ORR) using the best overall response after ipilimumab plus nivolumab treatment, which was defined as the percentage of patients who achieved complete response (CR) or partial response (PR) according to the RECIST version1.1 and iRECIST [15,16]. Follow-up CT for detecting any findings suspected of disease progression was scheduled every six weeks during the follow-up. Re-evaluation using magnetic resonance imaging (MRI), bone scintigraphy, and positron emission tomography/computed tomography (PET/CT) was adopted when necessary for the definitive diagnosis of immune-confirmed disease progression.

Ipilimumab plus nivolumab were administrated as follows: in the induction phase, ipilimumab was administered intravenously at a dose of 1 mg/kg for 30 min and nivolumab at 240 mg for 60 min, every three weeks for four times, followed by nivolumab monotherapy at a dose of 480 mg every four weeks as the maintenance phase. Discontinuation of nivolumab due to the disease progression or treatment-related AE was decided at the physician’s discretion. 

Serum calcium level, CRP, neutrophil-lymphocyte ratio (NLR), platelet-lymphocyte ratio (PLR), and hemoglobin were assessed in the prediction of lethality by adopting receiver operating characteristic (ROC) curve analysis. Harrell’s C-index was utilized to assess the prediction models [17]. The optimal cut-off values were defined by the Youden Index as the point maximizing the difference between the true-positive and false-positive rates for all possible cut-point levels [18,19]. The distribution of factors was analyzed by contingency table using Chi-square analysis. Student’s *t*-test and/or one-way analysis of variance (ANOVA) was used to assess the difference between the normal distributed variables. For variables with non-normal distribution, the Wilcoxon and/or Kruskal–Wallis test was conducted to assess the difference. A Kaplan–Meier curves were used to estimate the survival-free duration, and the log-rank test was performed to compare the difference between assigned patient groups. In the uni- and multivariate analyses, Cox proportional hazard regression models were utilized to determine crude hazard ratios (HRs) followed by the calculation of the covariate-adjusted HR. All the statistical tests were two-sided, with a *p*-value lower than 0.05 considered to indicate statistical significance. All analyses were conducted by JMP 13 package (SAS Institute Inc., Cary, NC, USA).

## 3. Results

**Figure 1:**
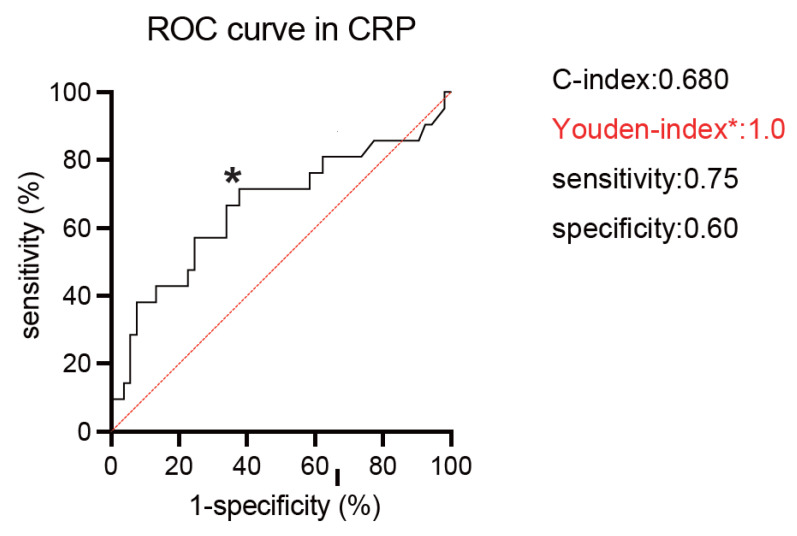
Receiver operating characteristic (ROC) curves of serum C-reactive protein (CRP: mg/dL) to predict lethality. The Youden index (* in the curve) identified a CRP of 1.0 mg/dL as an optimal cut-off value.

**Figure 2:**
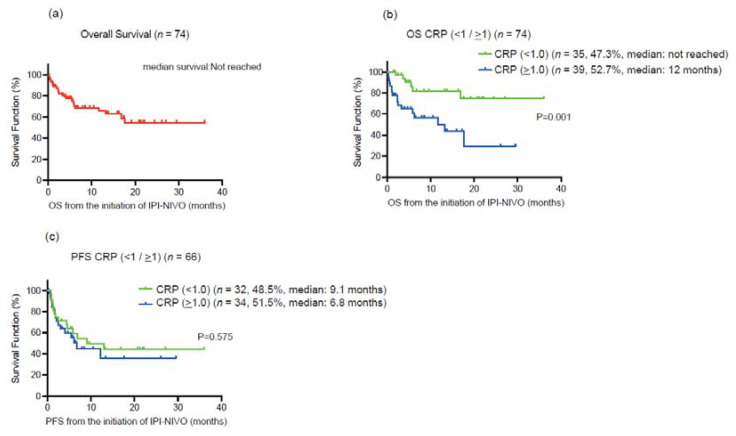
(**a**) Kaplan–Meier curve for overall survival (OS) in all 74 metastatic renal cell carcinoma (mRCC) patients treated with ipilimumab plus nivolumab. (**b**) Kaplan–Meier curves for overall survival (OS) according to the cut-off of serum C-reactive protein (CRP) 1.0 mg/dL. (**c**) Kaplan–Meier curves for progression-free survival overall survival (PFS) according to the cut-off of serum C-reactive protein (CRP) 1.0 mg/dL.

For all 74 patients, the median age was 68 years, and 24 (32.4%) patients were deceased during the follow-up. A median of 3.5 treatment cycles was performed, and 37 (50%) of patients completed four cycles of ipilimumab. A median follow-up was 6.5 months. Forty-five (61%) and 29 (39%) patients were diagnosed with IMDC intermediate and poor-risk groups, respectively. We first investigated the prognostic value for the lethality among putative biomarkers in blood examination (CRP, NLR, hemoglobin, PLR, corrected calcium) for mRCC patients treated with ipilimumab and nivolumab (Table 1). Of them, serum CRP showed the highest c-index of 0.680, and the Youden-index, which represents the cut-off value of serum CRP maximizing the difference between the true-positive and false-positive rates, was 1.0 mg/dL (Figure 1). We divided the cohort according to the 1.0 mg/dL of serum CRP level (Table 2). Patients with >1.0 mg/dL of CRP (39 patients) were more likely to be elder (*p* = 0.0499), undergo the prior nephrectomy (*p* < 0.001), and be classified to poor risk group (*p* < 0.001) compared to those with <1.0 mg/dL of CRP (35 patients). Kaplan–Meier curves exhibited a longer OS in patients with >1.0 mg/dL of CRP than those with <1.0 mg/dL (HR: 3.85, 95% CI: 1.68–8.79, *p* = 0.001), as well as CSS (HR: 3.42, 95% CI: 1.44–8.14, *p* = 0.006) (Figure 2). There was no significant difference in PFS between patients with CRP of <1.0 mg/dL and >1.0 mg/dL (*p* = 0.575). For the ORR in 74 patients, ORR and disease control rate (DCR) were 40.5% and 59.5%, respectively (Table 3).

**Figure 3:**
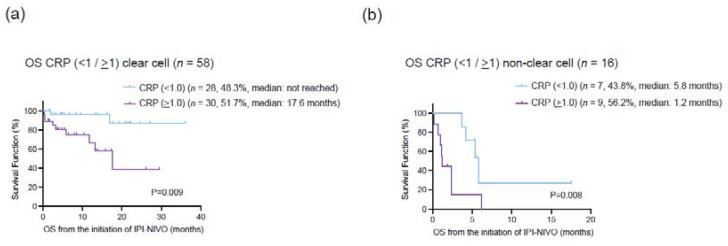
(**a**) Kaplan–Meier curves for overall survival (OS) in 58 clear cell renal cell carcinoma (ccRCC) according to the cut-off of serum C-reactive protein (CRP) 1.0 mg/dL. (**b**) Kaplan–Meier curves for overall survival (OS) in 16 non-clear cell RCC (nccRCC) according to the cut-off of serum C-reactive protein (CRP) 1.0 mg/dL.

To evaluate the prognostic value of serum CRP in mRCC patients treated with ipilimumab and nivolumab, we utilized stepwise cox regression analysis (Table 4). In univariate analysis, histological subtype (HR: 6.59, 95% CI: 2.8–15.4, *p* < 0.001), liver metastasis at the initiation of ipilimumab and nivolumab (HR: 2.88, 95% CI: 1.07–7.78, *p* = 0.037), and serum CRP at the initiation of ipilimumab and nivolumab (HR: 4.14, 95% CI: 1.63–10.5, *p* = 0.003) were correlated with OS. In multivariate analysis with these putative prognostic factors, histological subtype (HR: 7.89, 95% CI: 3.18–19.7, *p* < 0.001) and serum CRP at the initiation of ipilimumab and nivolumab (HR: 4.86, 95% CI: 1.83–12.9, *p* = 0.002) remained as independent predictors for OS. We also conducted the multivariate analysis for CSS and confirmed that histological type and serum CRP were independently associated with CSS. To further explore the prognostic impact on OS between histological subtype and serum CRP level, we examined Kaplan–Meier curves according to serum CRP (<1 and >1 mg/dL) stratified by the histological subtypes (ccRCC and non-ccRCC). OS for patients with CRP > 1 mg/dL was significantly shorter than those with CRP < 1 mg/dL in both ccRCC (a median OS of ‘not reached’ and 17.6 months, *p* = 0.009) and non-ccRCC (a median OS of 5.8 and 1.2 months, *p* = 0.008) (Figure 3).

## 4. Discussion

Serum CRP level have been analyzed as one of the systemic inflammation markers, which is associated with mRCC prognosis. In localized RCC, several reports further suggested the prognostic utility of CRP level with the combined assessment of neutrophil-lymphocyte ratio (NLR), platelet count, and albumin level for localized RCC treated with nephrectomy [7,9,13]. Ishihara et al. reported that the early change of serum CRP could serve as a predictive marker for mRCC patients treated with single-agent nivolumab therapy [10]. Most recently, several studies from the Japanese population showed the prognostic value of serum CRP for patients treated with first-line ipilimumab plus nivolumab treatment [11,12]. In other cancer types, serum CRP was proposed as a predictive marker of the treatment response of checkpoint inhibitors in hepatocellular carcinoma [20] and melanoma [21,22]. In the present study, we investigated the prognostic value of serum CRP level for mRCC patients treated with ipilimumab and nivolumab as the first-line treatment. Our clinical cohort was comprised of 58 ccRCC and 16 non-ccRCC patients. Serum CRP of 1 mg/dL was an ideal cut-off to discriminate the OS, and a higher serum CRP level was associated with shorter OS. In the CheckMate 214 trial, the median age was 62 years in 425 mRCC patients (IMDC risk group: 79% in intermediate and 21% in the poor-risk group) treated with ipilimumab and nivolumab with 12 and 18 months OS rate of 80% and 75%, respectively. The present study exhibited that the median age was 68 years in 74 mRCC patients (IMDC risk group: 61% in intermediate and 39% in the poor-risk group) with 12 and 18 months OS rates of 65% and 54%, respectively. Thus, the cohort in the present study from real-world practice represents elder and more aggressive tumor profiles than the CheckMate 214 trial. Nonetheless, given that the ORR in the present study was 40.5% being similar to 42% in the CheckMate 214 trial, the treatment with ipilimumab and nivolumab consistently offers clinical benefits in patients who were likely to be excluded from the benchmark trials.

Regarding the utility of serum CRP level for patients treated with ICI + tyrosine kinase inhibitors (TKIs), Tomita et al. recently reported the association of CRP with the efficacy of avelumab plus axitinib in mRCC using the long-term follow-up results from JAVELIN Renal 101 trial [23]. In their analysis, patients were divided into three groups, including normal (baseline CRP < 1 mg/dL) in 234 patients, normalized (baseline CRP > 1 mg/dL and decreased to <1 mg/dL during 6-week treatment) in 51 patients, and non-normalized (CRP > 10 mg/l at baseline and during 6-week treatment) in 108 patients. ORR was 56.0% in normal, 66.7% in normalized, and 45.4% in non-normalized groups with avelumab plus axitinib treatment. Median PFS was 15.2 months in normal, not reached (NR) in normalized, and 7.0 months in non-normalized groups. With the multivariate analysis showing normalized CRP as an independent predictor for favorable objective response and OS, they concluded that serum CRP levels at baseline might predict efficacy with avelumab plus axitinib. Klumper et al. also reported the utility of CRP kinetics for patients treated with ICI + ICI (59 patients) and ICI + TKI (39 patients) treatment for mRCC. In their analysis, early CRP kinetics was significantly associated with improved PFS in both ICI + ICI and ICI + TKI subgroups [24].

nccRCC includes various histological patterns, such as papillary, chromophobe, adenocarcinoma, translocation unclassified, medullary, and collecting duct RCC, which roughly accounts for 25% of kidney cancer [25]. In a retrospective report from the IMDC, patients with nccRCC were associated with worse outcomes compared with those with ccRCC (median survival of 12.8 and 22.3 months in nccRCC and ccRCC, respectively) [26]. Due to the lack of prospective evidence for the treatment of nccRCC, treatment strategy has often been extrapolated from the result of benchmark randomized trials of ccRCC. Indeed, the CheckMate 214 trial only recruited ccRCC patients. Recently, several reports have shown treatment activity with the first-line ipilimumab and nivolumab in nccRCC. Gupta et al. reported the ORR of 50% (CR: 16.7% and PR: 33.3%) for 18 nccRCC patients [27]. Tykodi et al. recently reported the results from the phase 3b/4 CheckMate 920 trial that involved 52 nccRCC patients with 19.6% (95% CI: 9.4–33.9) of ORR [28]. Currently, a randomized phase-2 study of ipilimumab and nivolumab vs. standard of care in untreated and advanced nccRCC is ongoing (NCT03075423). In the present study, our multivariate analysis exhibited that nccRCC and the increased serum CRP level were independent prognostic indicators of the poor OS. Importantly, a higher CRP level was associated with shorter OS in ccRCC and nccRCC, suggesting the prognostic value of serum CRP for the first-line ipilimumab and nivolumab in mRCC including nccRCC.

Several limitations should be clarified in the present study, including its retrospective nature with shorter follow-up duration and relatively small sample size. In addition, we could not incorporate the examination of genetic alternations and molecular characterizations, such as PD-L1 expression and tumor mutation burden. Lastly, an elevation of serum CRP is not specific to mRCC. The cohort in the present study does not include information on detailed complications or past history that can be affected to the serum CRP level, such as inflammatory bowel disease, lupus, rheumatoid arthritis, and infections. Further assessment in a larger and prospective study is needed to validate our findings.

## 5. Conclusions

Serum CRP level is a prognostic indicator for OS in both ccRCC and nccRCC patients treated with the first-line ipilimumab plus nivolumab treatment.

## Figures and Tables

**Table 1:** Comparison of C-index among serum biomarkers for predicting the lethality in mRCC treated by IPI plus NIVO.

	C-Index	Youden-Index
CRP	0.680	1.0
PLR	0.658	254.0
Ca	0.618	9.9
Hb	0.618	10.5
NLR	0.615	5.7

mRCC: metastatic renal cell carcinoma, IPI: ipilimumab, NIVO: nivolumab, CRP: C-reactive protein, PLR: platelet-to-lymphocyte ratio, Ca: calcium, Hb: hemoglobin, NLR: neutrophil-to-lymphocyte ratio.

**Table 2:** Patient characteristics at the nitiation of ipilimumab plus nibolumab in 74 mRCC patients.

	Total	CRP mg/dL <1.0	CRP mg/dL ≥1.0	
Clinical Variables	*n* = 74	*n* = 35	*n* = 39	*p* Value
Age (mean ± SD)	63.7 ± 11.3	61.2 ± 11.7	65.9 ± 10.7	0.050
<70	50 (67.6)	28 (80.0)	21 (53.9)	
≥70	24 (32.4)	7 (20.0)	18 (46.2)	0.026 *
Sex (%)				
male	58 (78.4)	25 (71.4)	33 (84.6)	
female	16 (21.6)	10 (28.6)	6 (15.4)	0.258
BMI (%)				
<25	56 (75.7)	28 (80.0)	28 (71.8)	
≥25	18 (24.3)	7 (20.0)	11 (28.2)	0.433
IMDC risk classification (%)				
intermediate	45 (61.0)	31 (88.6)	14 (35.9)	
poor	29 (39.0)	4 (11.4)	25 (64.1)	<0.001 *
Prior nephrectomy before IPI plus NIVO (%)				
no	37 (50.0)	9 (25.7)	28 (71.8)	
yes	37 (50.0)	26 (74.3)	11 (28.2)	<0.001 *
Pathological type of tumor (%)				
clear cell carcnoma	58 (78.4)	28 (80.0)	30 (77.0)	
not clear cell carcinoma	16 (21.6)	7 (20.0)	9 (23.0)	0.785
Location of metastatic sites at the initiation of IPI plus NIVO (%)				
Lung	45 (60.8)	22 (62.9)	23 (59.0)	0.814
Liver	8 (10.8)	2 (5.7)	6 (10.8)	0.267
Lympho node	26 (35.1)	8 (22.9)	18 (46.1)	0.051
irAEs during follow–up (%)				
0–2	54 (73.0)	25 (71.4)	29 (74.4)	
≥3	20 (27.0)	10 (28.6)	10 (25.6)	0.799
PLR at the initiation of IPI plus NIVO (median: [IQR])	205 [151, 308]	162 [124, 208]	254 [180, 440]	<0.001 *
NLR at the initiation of IPI plus NIVO (median: [IQR])	3.81 [2.75, 6.15]	2.94 [2.2, 4.32]	4.88 [3.65, 7.5]	0.038 *
Hemogulobin level at the initiation of IPI plus NIVO (median: [IQR])	11.4 [9.68, 12.9]	12.3 [11, 13.7]	10.2 [8.2, 11.5]	<0.001 *
Eorrected calcium level at the initiation of IPI plus NIVO	9.8 [9.4, 10.3]	9.6 [9.1, 9.8]	10.3 [9.8, 10.9]	<0.001 *

mRCC: metastatic renal cell carcinoma, CRP: C-reactive protein, SD: standard deviation, IPI: ipilimumab, NIVO: nivolumab, BMI: body mass index, IMDC: international metastatic RCC database consortium, irAE: immne-related adverse event, PLR: platelet-lymphocyte ratio, * denotes *p* < 0.05.

**Table 3:** Confirmed best objective response for 74 patients treated with ilipimumab and nivolumab.

		CRP mg/dL <1.0	CRP mg/dL ≥1.0
Variable	*n* = 74	*n* = 35	*n* = 39
ORR (%)	40.5	31.4	48.7
DCR (%)	59.5	65.7	53.8
Confirmed best overall response—no. (%)			
CR	4 (5.4)	2 (5.7)	2 (5.1)
PR	26 (35.1)	9 (25.7)	17 (43.6)
SD	14 (18.9)	12 (34.8)	2 (5.1)
PD	16 (21.6)	7 (20.0)	9 (23.1)
Unable to determine or not reported	14 (18.9)	5 (14.3)	9 (23.1)

CRP: C-reactive protein, ORR: objective response rate, DCR: disease control rate, CR: complete response, PR: partial response, SD: stable disease, PD: progression disease.

**Table 4:** Univariate and Multivariate analyses for predicting OS and CSS in mRCC treated by IPI plus NIVO.

	OS						CSS					
Clinical Variables	Univariable			Multivariable			Univariable			Multivariable		
	HR	(95% CI)	*p*-Value	HR	(95% CI)	*p*-Value	HR	(95% CI)	*p*-Value	HR	(95% CI)	*p*-Value
Age at the initiation of IPI plus NIVO (years)												
(<70/≥70)	1.16	0.46–2.93	0.751				1.29	0.52–3.21	0.583			
Gender												
(Male/Female)	1.54	0.28–0.67	0.305				1.39	0.53–3.58	0.499			
BMI at the initiation of IPI plus NIVO												
(<25/≥25)	1.08	0.34–0.40	0.877				1.08	0.39–2.97	1.08			
Prior nephrectomy before IPI plus NIVO												
(no/yes)	1.48	0.66–3.30	0.341				1.38	0.59–3.26	0.459			
Histological type of tumor												
(clear cell carcnoma/not clear cell carcnoma)	6.59	2.80–15.4	<0.001 *	7.89	3.18–19.7	<0.001 *	6.95	2.78–17.37	<0.001 *	8.40	3.12–22.6	<0.001 *
Liver metastasis at the initiation of IPI plus NIVO												
(−/+)	2.88	1.07–7.78	0.037	1.74	0.59–5.08	0.313	3.52	1.28–9.73	0.015	2.14	0.71–6.49	0.179
Lung metastasis at the initiation of IPI plus NIVO												
(−/+)	1.26	0.55–2.87	0.590				1.53	0.62–3.79	0.361			
Immune related a during follow-up												
(0–2/≥3)	1.00	0.42–2.44	0.982				1.22	0.49–3.02	0.674			
CRP at the initiation of IPI plus NIVO												
(<1.0/≥1.0)	4.14	1.63–10.5	0.003 *	4.86	1.83–12.9	0.002	3.53	1.35–9.20	0.001 *	4.15	1.50–11.5	0.006

mRCC: mRCC: metastatic renal cell carcinoma, IPI: ipilimumab, NIVO: nivolumab, BMI: body mass index, IMDC: international metastatic RCC database consortium, CRP: C-reactive protein, OS: overrall survival, CSS: canser specific survival, HR: hazard ratio, CI: Confidence interval, * denotes *p* < 0.05.

## Data Availability

Not applicable.

## References

[1] Choueiri T.K., Powles T., Burotto M., Escudier B., Bourlon M.T., Zurawski B., Oyervides Juarez V.M., Hsieh J.J., Basso U., Shah A.Y. (2021). Nivolumab plus Cabozantinib versus Sunitinib for Advanced Renal-Cell Carcinoma. N. Engl. J. Med..

[2] Motzer R., Alekseev B., Rha S.Y., Porta C., Eto M., Powles T., Grunwald V., Hutson T.E., Kopyltsov E., Mendez-Vidal M.J. (2021). Lenvatinib plus Pembrolizumab or Everolimus for Advanced Renal Cell Carcinoma. N. Engl. J. Med..

[3] Motzer R.J., Penkov K., Haanen J., Rini B., Albiges L., Campbell M.T., Venugopal B., Kollmannsberger C., Negrier S., Uemura M. (2019). Avelumab plus Axitinib versus Sunitinib for Advanced Renal-Cell Carcinoma. N. Engl. J. Med..

[4] Rini B.I., Plimack E.R., Stus V., Gafanov R., Hawkins R., Nosov D., Pouliot F., Alekseev B., Soulieres D., Melichar B. (2019). Pembrolizumab plus Axitinib versus Sunitinib for Advanced Renal-Cell Carcinoma. N. Engl. J. Med..

[5] Motzer R.J., Tannir N.M., McDermott D.F., Aren Frontera O., Melichar B., Choueiri T.K., Plimack E.R., Barthelemy P., Porta C., George S. (2018). Nivolumab plus Ipilimumab versus Sunitinib in Advanced Renal-Cell Carcinoma. N. Engl. J. Med..

[6] Motzer R.J., Choueiri T.K., McDermott D.F., Powles T., Vano Y.A., Gupta S., Yao J., Han C., Ammar R., Papillon-Cavanagh S. (2022). Biomarker analysis from CheckMate 214: Nivolumab plus ipilimumab versus sunitinib in renal cell carcinoma. J. Immunother. Cancer.

[7] Komura K., Hashimoto T., Tsujino T., Muraoka R., Tsutsumi T., Satake N., Matsunaga T., Yoshikawa Y., Takai T., Minami K. (2019). The CANLPH Score, an Integrative Model of Systemic Inflammation and Nutrition Status (SINS), Predicts Clinical Outcomes After Surgery in Renal Cell Carcinoma: Data From a Multicenter Cohort in Japan. Ann. Surg. Oncol..

[8] Ramsey S., Lamb G.W., Aitchison M., Graham J., McMillan D.C. (2007). Evaluation of an inflammation-based prognostic score in patients with metastatic renal cancer. Cancer.

[9] Tsujino T., Komura K., Ichihashi A., Tsutsumi T., Matsunaga T., Yoshikawa Y., Maenosono R., Okita K., Takai T., Oide R. (2017). The combination of preoperative platelet count and neutrophil lymphocyte ratio as a prognostic indicator in localized renal cell carcinoma. Oncotarget.

[10] Ishihara H., Takagi T., Kondo T., Fukuda H., Tachibana H., Yoshida K., Iizuka J., Okumi M., Ishida H., Tanabe K. (2020). Predictive impact of an early change in serum C-reactive protein levels in nivolumab therapy for metastatic renal cell carcinoma. Urol. Oncol..

[11] Tachibana H., Nemoto Y., Ishihara H., Fukuda H., Yoshida K., Iizuka J., Hashimoto Y., Kondo T., Tanabe K., Takagi T. (2022). Predictive Impact of Early Changes in Serum C-reactive Protein Levels in Nivolumab Plus Ipilimumab Therapy for Metastatic Renal Cell Carcinoma. Clin. Genitourin. Cancer.

[12] Tanaka T., Hatakeyama S., Numakura K., Kido K., Noro D., Oikawa M., Hosogoe S., Tokui N., Yamamoto H., Narita S. (2020). Efficacy and safety of first-line nivolumab plus ipilimumab in patients with metastatic renal cell carcinoma: A multicenter retrospective study. Int. J. Urol..

[13] Tsujino T., Komura K., Hashimoto T., Muraoka R., Satake N., Matsunaga T., Tsutsumi T., Yoshikawa Y., Takai T., Minami K. (2019). C-reactive protein-albumin ratio as a prognostic factor in renal cell carcinoma—A data from multi-institutional study in Japan. Urol. Oncol..

[14] Heng D.Y., Xie W., Regan M.M., Warren M.A., Golshayan A.R., Sahi C., Eigl B.J., Ruether J.D., Cheng T., North S. (2009). Prognostic factors for overall survival in patients with metastatic renal cell carcinoma treated with vascular endothelial growth factor-targeted agents: Results from a large, multicenter study. J. Clin. Oncol..

[15] Eisenhauer E.A., Therasse P., Bogaerts J., Schwartz L.H., Sargent D., Ford R., Dancey J., Arbuck S., Gwyther S., Mooney M. (2009). New response evaluation criteria in solid tumours: Revised RECIST guideline (version 1.1). Eur. J. Cancer.

[16] Seymour L., Bogaerts J., Perrone A., Ford R., Schwartz L.H., Mandrekar S., Lin N.U., Litière S., Dancey J., Chen A. (2017). iRECIST: Guidelines for response criteria for use in trials testing immunotherapeutics. Lancet Oncol..

[17] Harrell F.E., Lee K.L., Mark D.B. (1996). Multivariable prognostic models: Issues in developing models, evaluating assumptions and adequacy, and measuring and reducing errors. Stat. Med..

[18] Unal I. (2017). Defining an Optimal Cut-Point Value in ROC Analysis: An Alternative Approach. Comput. Math. Methods Med..

[19] Youden W.J. (1950). Index for rating diagnostic tests. Cancer.

[20] Zhang Y., Lu L., He Z., Xu Z., Xiang Z., Nie R.C., Lin W., Chen W., Zhou J., Yin Y. (2022). C-reactive Protein Levels Predict Responses to PD-1 Inhibitors in Hepatocellular Carcinoma Patients. Front. Immunol..

[21] Yoshida T., Ichikawa J., Giuroiu I., Laino A.S., Hao Y., Krogsgaard M., Vassallo M., Woods D.M., Stephen Hodi F., Weber J. (2020). C reactive protein impairs adaptive immunity in immune cells of patients with melanoma. J. Immunother. Cancer.

[22] Laino A.S., Woods D., Vassallo M., Qian X., Tang H., Wind-Rotolo M., Weber J. (2020). Serum interleukin-6 and C-reactive protein are associated with survival in melanoma patients receiving immune checkpoint inhibition. J. Immunother. Cancer.

[23] Tomita Y., Larkin J., Venugopal B., Haanen J., Kanayama H., Eto M., Grimm M.O., Fujii Y., Umeyama Y., Huang B. (2022). Association of C-reactive protein with efficacy of avelumab plus axitinib in advanced renal cell carcinoma: Long-term follow-up results from JAVELIN Renal 101. ESMO Open.

[24] Klumper N., Schmucker P., Hahn O., Hoh B., Mattigk A., Banek S., Ellinger J., Heinzelbecker J., Sikic D., Eckstein M. (2021). C-reactive protein flare-response predicts long-term efficacy to first-line anti-PD-1-based combination therapy in metastatic renal cell carcinoma. Clin. Transl. Immunol..

[25] Ferlay J., Soerjomataram I., Dikshit R., Eser S., Mathers C., Rebelo M., Parkin D.M., Forman D., Bray F. (2015). Cancer incidence and mortality worldwide: Sources, methods and major patterns in GLOBOCAN 2012. Int. J. Cancer.

[26] Kroeger N., Xie W., Lee J.L., Bjarnason G.A., Knox J.J., Mackenzie M.J., Wood L., Srinivas S., Vaishamayan U.N., Rha S.Y. (2013). Metastatic non-clear cell renal cell carcinoma treated with targeted therapy agents: Characterization of survival outcome and application of the International mRCC Database Consortium criteria. Cancer.

[27] Gupta R., Ornstein M.C., Li H., Allman K.D., Wood L.S., Gilligan T., Garcia J.A., Merveldt D.V., Hammers H.J., Rini B.I. (2020). Clinical Activity of Ipilimumab Plus Nivolumab in Patients with Metastatic Non-Clear Cell Renal Cell Carcinoma. Clin. Genitourin. Cancer.

[28] Tykodi S.S., Gordan L.N., Alter R.S., Arrowsmith E., Harrison M.R., Percent I., Singal R., Van Veldhuizen P., George D.J., Hutson T. (2022). Safety and efficacy of nivolumab plus ipilimumab in patients with advanced non-clear cell renal cell carcinoma: Results from the phase 3b/4 CheckMate 920 trial. J. Immunother. Cancer.

